# Developmental Eye Movement (DEM) and King-Devick (K-D) Performance in Multiple Sclerosis

**DOI:** 10.3390/brainsci12070954

**Published:** 2022-07-20

**Authors:** Amparo Gil-Casas, David P. Piñero-Llorens, Ainhoa Molina-Martín

**Affiliations:** 1 Clínica Optomètrica, Foundation Lluís Alcanyís, University of Valencia, 46020 Valencia, Spain; amparo.gil@fundacions.uv.es; 2 Optics and Visual Perception Group (GOPV), Department of Optics, Pharmacology and Anatomy, University of Alicante, 03690 San Vicent del Raspeig, Spain; david.pinyero@ua.es

**Keywords:** multiple sclerosis, DEM, K-D test, saccades, eye movement, reading

## Abstract

Eye movement disorders have been reported in patients with multiple sclerosis (MS) as saccadic disturbances. Several methods have been described for the assessment of saccades, including the K-D and DEM tests. The performance of these tests also involves attention, language, and other brain areas which have been reported to be altered in MS patients. The aim of the study was to determine how developmental eye movement (DEM) and King-Devick (K-D) tests are affected in MS-patients. It was also to analyze whether a resolved episode of optic neuritis (ON) has a negative influence. Subjects with MS showed worse outcomes (higher times) in DEM test (72 (26) s and a K-D test (56 (22) s compared to healthy subjects (64 (7) s and 55 (11) s, respectively). However, a previous ON episode did not worsen the MS-times of DEM (80 (33) s or of K-D (62 (33) s. Horizontal saccades with the DEM showed differences between subjects with MS (with and without ON) and healthy ones (*p* < 0.05), whereas no such differences were found in the vertical saccades. According to the DEM instructions, MS patients would present heterogeneous oculomotor and non-visual disturbances. Regarding the K-D test, only the third card (the most complex one) showed differences (*p* < 0.05) between groups. These tests can capture impairment of attention, language, and other areas that correlate with suboptimal brain function in addition to the oculomotor dysfunctions present in subjects with MS.

## 1. Introduction

Ocular motor dysfunction is present in almost 90–95% of the patients with multiple sclerosis (MS) due to the demyelination in the visual pathway [1,2]. Demyelinating lesions in the posterior fossa are a frequent cause of ocular motor dysfunction [3]. Saccades disorders, such as saccadic dysmetria, and impaired smooth pursuit are the most common dysfunctions among MS subjects [4,5]. The measurement of saccades has been proposed as a biomarker of the disease stage, progression, and response to therapy in neurodegenerative diseases [6].

Additionally, most patients of MS reported symptomatology such as blurring vision, oscillopsia, asthenopia and reduced depth perception, which are related to oculomotor disorders [7,8,9]. Binocular vision has also been found to be affected in MS patients [10,11]. These alterations cause impaired vision in MS patients, which can result in a significant reduction in their quality of life [12].

There are several methods to assess eye movements. The conventional method is observation, grading fixation stability, pursuit, and saccadic eye movements subjectively by the examiner. However, other more reliable and repeatable methods such as microperimetry or video-oculography have demonstrated its utility in eye movement analysis [13], even in MS patients [14]. Moreover, eye movements can be measured indirectly during tasks that simulate reading, such as the King Devick test (K-D) [15] and the developmental Eye Movement Test (DEM) [16,17].

The King Devick test (K-D) was originally designed to assess reading ability [15], and evaluates processing speed and visual tracking, which require saccadic eye movements, attention, and language function. Its use has been extended to the detection of various neurological conditions, such as Parkinson’s disease [18], Alzheimer’s disease [19] and amyotrophic lateral sclerosis [20]. MS patients showed higher K-D times which were related to greater disability and worse quality of life measured with the 25-Item National Eye Institute Visual Functioning Questionnaire (NEI-VFQ-25) [21].

On the other hand, the Developmental Eye Movement Test (DEM) was designed to assess reading performance and visual processing speed in children [16,17]. Its correlation with saccadic eye movement skills and symptomatology has been questioned [22]. To the best of our knowledge, the DEM test has not been administered to MS patients in any previous study nor to any patients with any neurodegenerative disease; it is therefore unknown how these neurodegenerative diseases affect the performance of the test.

The aim of the present study was to evaluate the reading performance of subjects with MS through K-D and DEM tests and to compare the results with those obtained in healthy subjects. Furthermore, as episodes of optic neuritis (ON) are common in MS [18], a group with a previous ON was set up to assess whether ON affects the performance ability on those tests.

## 2. Materials and Methods

### 2.1. Subjects

Patients with MS were recruited from two centers: the Optometry Clinic of Foundation Lluís Alcanyís of the University of Valencia and the Optometry Clinic of the University of Alicante. All participants signed written informed consent according to the Declaration of Helsinki, and the study protocol was previously approved by the Ethics Committee of the University of Alicante and University of Valencia. The inclusion criterion was the presence of MS, but the degree of disability and type of MS were not considered when setting up the study groups. However, the majority of patients exhibited relapsing-remitting MS (RRMS). Exclusion criteria were the presence the comorbidity with other ocular or systemic diseases that could affect the study results (such as cataract or muscular paralysis). Results were compared to those obtained in a sample of healthy controls without ocular findings matched by age and sex.

### 2.2. Clinical Procedure

A complete visual examination was performed on all subjects, and included manifest refraction, visual acuity (monocular and binocular), and measurement of heterophoria and stereopsis at near vision. Heterophoria was measured with the alternating Cover Test (CT) and the use of a prism bar to neutralize eye movements. The Randot Preschool Stereo Acuity Test (Stereo Optical Company, Chicago, IL, USA) was used to measure stereopsis. This test was administered according to the manufacturer’s instructions and was scored by its logarithmic transformation for statistical analysis purposes as suggested by Webber et al. [23] (i.e., a stereopsis value of 200 sec arc was scored as log200 = 2.30). In those subjects with null stereopsis, the Worth test was used to confirm the presence or not of simultaneous vision. If diplopia was present, a score of 4 was assigned to that subject, whereas if suppression was present a score of 5 was assigned.

Following this clinical examination, participants were randomly assigned to the sequence of testing with either the DEM or K-D test first. Both tests were administered by the same investigator (AGC). All examinations were performed with participants wearing their best corrected visual acuity with either spectacles or contact lenses for near vision. Subjects were instructed to perform the tests using the demonstration cards. Both tests were performed at near vision (40 cm) and binocularly.

#### 2.2.1. K-D Test

The King-Devick (K–D) test is based on measuring the time needed to perform a reading task, and consequently requires the integration of eye movements, language function and attention [24]. Participants should read three cards aloud, which are composed of a series of random single-digit numbers from left to right, as quickly as possible. The difficulty of the cards increases progressively due to changes in spacing between numbers and lines. The cumulative time spent in completing the three cards is defined as the K-D test summary score. The number of errors made while reading was also recorded.

#### 2.2.2. DEM Test

The DEM test is also based on the time to perform a reading task, but in contrast to the K-D test, DEM was developed to differenciate problems of verbalization by introducing the vertical component in the examination [25]. DEM test also includes three cards, the first two consist of two vertical columns with 40 single-digit numbers; the last one is comprised of the same numbers, but oriented horizontally in 16 rows of five single-digits with a different distance between the digits on each row. The time in performing the reading task of the three cards was recorded as time V1, time V2 and Time H. The adjusted horizontal time (Hadj) was calculated as: Hadj = time H × 80/(80 − O + A), being: O = omissions and A = additions. Additionally, the ratio between the horizontal adjusted time and the vertical time was also analyzed as: R = Hadj/VT, where VT is the sum of the times of the two vertical cards [16,26,27].

According to the value of the vertical and horizontal time, and the ratio, DEM’s instructions can classify the patients as: Type I (vertical and horizontal time, and ratio in normal limits) representing a normal performance; Type II (vertical time normal, but increased horizontal time and ratio) suggesting an oculomotor disturbance; Type III (both vertical and horizontal time increased, with normal ratio) suggesting a difficulty in visual-verbal skills; and Type IV (vertical and horizontal time, and ratio increased) suggesting both oculomotor disturbance and difficulties in visual-verbal skills.

### 2.3. Statistical Analysis

Statistical analysis of the results was done using the SPSS program v.26.0.0 for Windows (SPSS Inc., Chicago, IL, USA, Ill). None of the studied parameters followed a Normal distribution according to a Kolmogorov-Smirnov test, and non-parametric tests were applied. Differences between groups were assessed with the Kruskal-Wallis test for the parameters described above for K-D (time Card 1, 2 and 3, total time and errors) and DEM (time V1, V2, H, and errors, Hadj, VT, and ratio). Additionally, differences between groups were assessed for VA (binocular and difference between eyes), heterophoria and stereopsis. Correlation between clinical parameters and the K-D and DEM variables was assessed by Spearman correlation coefficient. Differences in the frequency of classification of DEM in subgroups was assessed by Chi-square test.

## 3. Results

A total of 85 patients were recruited and divided into three groups. MS groups consisted of 35 subjects in the group without ON and 30 in the group with previous ON (not active in the moment of examination). The control group consisted of 20 healthy subjects. Clinical and demographic characteristics of the three study groups are provided in Table 1.

Differences in VA between eyes (ΔVA) did not differ between groups. Regarding binocular VA, all three groups achieve good VA, close to 0.00 log MAR, better in the control group but with no statistically significant differences between groups (*p* = 0.06). Heterophoria measurement of the three groups showed a negative heterophoria (exophoria) for near vision, being lower in the MSON group but with no significant differences (*p* = 0.42). In contrast, there were significant differences in Stereopsis between the control group and the MS group (*p* < 0.001), and with MSON group (*p* < 0.001), but not among the sclerosis groups (*p* = 0.18).

### 3.1. K-D Test

The results of the K-D test outcomes (times and errors) for each card are shown in Table 2. MSON was the group that took the longest time with a median value (and interquartile range) of 62 (33) s, followed by the MS group with 56 (22) s, and the control group with 55 (11) s. The time used to perform the entire K-D test showed differences between groups (*p* < 0.05). However, there were differences only between the control group and the MS and MSON groups (*p* = 0.04 and 0.03 respectively), but not between the MS and MSON groups (*p* = 0.42).

**Figure 1:**
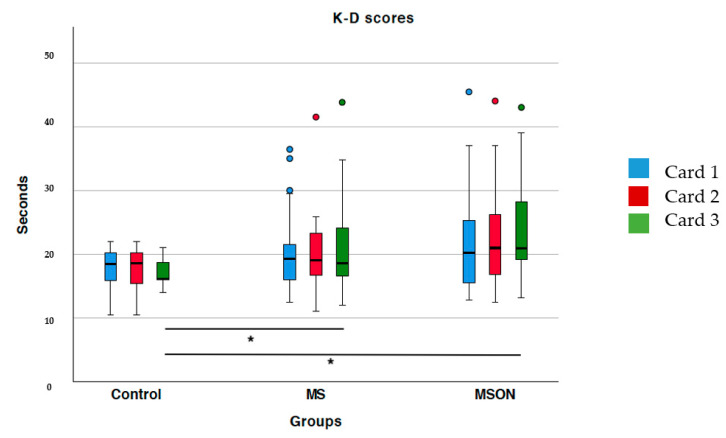
2 and 3 of the K-D test per groups. Statistically significant differences were marked with an asterisk (*).

If the three cards are analyzed individually, only the control group was differentiated from the two multiple sclerosis groups in card 3 (*p* < 0.05 in both cases). However, the two MS groups showed no statistically significant differences between them (*p* = 0.26) in that card (Figure 1). On the other hand, the times for the first two cards were similar between the three groups. (*p* > 0.05). Although the sclerosis groups made more errors than the control group, there were no statistically significant differences in either letter (*p* > 0.05).

The MSON group showed a weak but significant correlation of stereopsis with: time needed to read the first card (r = 0.46, *p* = 0.01); the second card (r = 0.38, *p* = 0.03); and total time (r = 0.39, *p* = 0.03). For all other clinical parameters analyzed (heterophoria and VA), there was no correlation. In the other two groups (control and MS), no significant correlations were observed.

### 3.2. DEM Test

The DEM test outcomes (times and errors) are shown in Table 3. The group that took the longest median time to complete all three cards, and thus the total time, was the MSON group with a median value (and interquartile range) of 80 (33) s, followed by the MS group with 72 (26) s, and the control group with 64 (7) s. The analysis of differences by pairs revealed the presence of statistically significant differences in the total time only between the MSON and control group (*p* < 0.001), and close to significance (*p* = 0.06) between control and MS group, but not among the sclerosis groups (*p* = 0.69).

**Figure 2:**
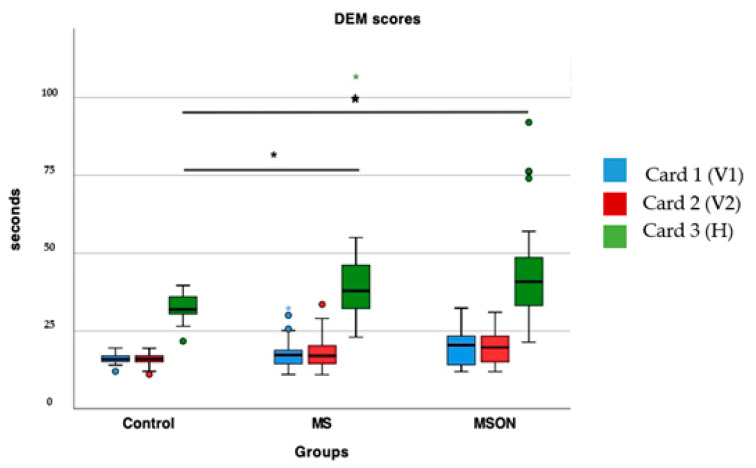
Time spent in completing the cards 1, 2 and 3 of DEM test per group. Cards 1 and 2 were read vertically with different combinations of numbers and separations (V1 and V2), whereas card 3 was read horizontally (H). Statistically significant differences are marked with an asterisk (*).

The control group showed statistically significant shorter times on the horizontal card compared to the MS group (*p* = 0.03) and with MSON (*p* = 0.02), as can be seen in Figure 2. However, the two sclerosis groups did not differ in the time spent on that card (*p* = 0.23). On the other hand, the times of vertical cards were similar among the three groups (*p* > 0.05). Regarding the errors, all three groups showed similar errors in all three cards (*p* > 0.05).

Regarding the DEM ratio, higher values (which can represent normal vertical times with horizontal times increased, or an increase in both components but more in the horizontal component) were found in the MS group, these differences being statistically significant in comparison to the control group (*p* < 0.001), but not to the MSON (*p* = 0.52). In contrast, the MSON group did not show significant differences with the control group (*p* = 0.42) in the ratio, since both vertical and horizontal times were longer than in the control group.

No correlations were found between DEM outcomes and clinical parameters (heterophoria, VA and stereopsis) in any group (*p* > 0.05 in all cases). On the other hand, there was a positive correlation between the vertical and horizontal times of the DEM test in all three groups: Control (r = 0.65, *p* = 0.00), MS (r = 0.89, *p* < 0.001) and MSON (r = 0.82, *p* < 0.001), that is, those subjects spending more time on the vertical card also take longer to read the horizontal card.

#### DEM Classification

**Figure 3:**
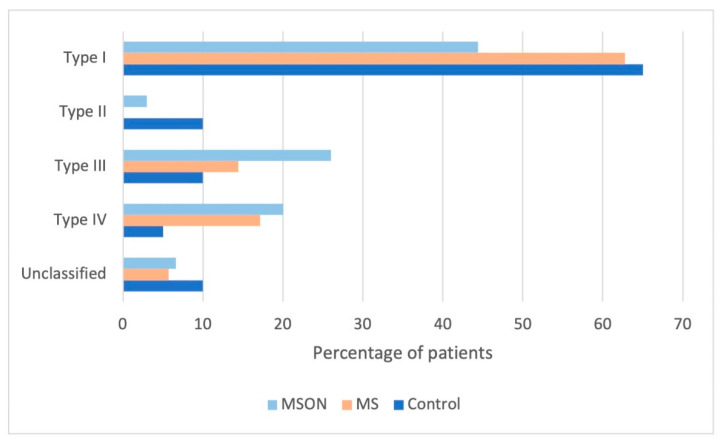
Percentage of the patients classified according to DEM instructions. MSON: group of multiple sclerosis with previous optic neuritis. MS: multiple sclerosis group.

According to the results, and the DEM instructions, MS patients were classified as follows: Type I (63% MS-patients); Type II (none of the subjects); Type III (14% of the MS-patients); Type IV (17% MS-patients). A lower-than-normal ratio (i.e., vertical times were more increased than horizontal times) was present in some subjects (6% of MS subjects), therefore an additional type was included and named as unclassified, since this assumption is not addressed in the interpretation of the DEM results (Figure 3).

These percentages were slightly different in the MS group with a previous episode of optic neuritis, with no statistical differences between groups in the frequency of classification (*p* > 0.05 in all cases). Approximately 43% of these patients showed Type I. Only 3% would be classified as Type II. Type III would account for 27% of the patients, and Type IV would be comprised of 20% of them. Seven percent of the patients could not classified according to the instructions.

## 4. Discussion

Visual function is essential to carrying out daily activities, such as reading or watching TV. In fact, the 25-Item National Eye Institute Visual Function Questionnaire (VFQ-25) revealed that visual function is among the most valued by MS patients [9]. To perform DEM and K-D tests correctly, visual functions such as fixation, saccades, and binocular coordination must be in good conditions [14,28,29], but also other abilities such as language skills and cognitive function. Therefore, pseudo-reading tests like the DEM and K-D test could provide information on visual and brain damage in MS patients.

### 4.1. K-D Test

The K-D test has been studied previously among MS patients and has been associated with neurological disability and reduced quality of life [28,30]. Poorer K-D results have been associated with alterations in saccades measured through video-oculography by other authors [5,31] in Alzheimer’s disease [19], sports-related concussion [32], sleep deprivation [33], and hypoxia [34]. Furthermore, the King-Devick test was significantly associated with deficits in low-contrast visual acuity and retinal nerve fiber layer (RNFL) thinning in MS patients [21].

Regarding the results obtained in the present work, the K-D times for the three groups were like those previously obtained by other authors [21,31,35,36]. MS subjects took longer to complete the whole K-D test, which could be associated with a greater difficulty in generating saccades, as other authors have observed [5,19,31,32]. In our case, as no quantifiable assessment of saccades was performed by objective methods, it is not possible to relate worse K-D scores to saccadic impairment. Moreover, MS subjects showed longer times on the third card, which is the most difficult one, because the numbers are represented with variable spacing and without the aid of lines connecting numbers. These results suggest that the more difficult the task required, the more time MS subjects take to complete it. Surprisingly, this complexity in the task is not related to a higher number of errors in the performance of the test, since the number of errors was similar in all three cards and did not significantly differ from healthy subjects. Furthermore, the K-D test can capture the impairment of eye movements, attention, language, and other areas that correlate with suboptimal brain function, so it is possible that MS patients also show disturbances in those capacities.

The history of ON has been associated with an increased K-D score [21], but the results of the current series did not show differences between sclerosis groups, with or without ON. In contrast, authors such as Moster et al. [21] found a relationship between ON and worse K-D scores and relied on binocular summation to justify these findings. Binocular summation is an improvement of binocular vision compared to monocular vision [37,38], and a decrease in binocular summation, or even binocular inhibition, has been found in patients with ON caused by the difference in VA between the two eyes [39]. In contrast to the sample analyzed by Moster et al. [21], in the present study, subjects from the MSON group had a previous and resolved ON in the moment of the examination, and differences between eyes in the VA were negligible compared to healthy subjects. Instead, the results of the present study showed that most of subjects exhibit some grade of stereopsis, demonstrating that binocular summation was present, and suggesting that a previous resolved episode of ON may not affect the results, as long as VA and binocular vision was restored.

### 4.2. DEM Test

Regarding the neural circuitry of vertical and horizontal saccades, it is postulated that the neural pathway which controls horizontal and vertical movement is different between horizontal and vertical [40,41]. In fact, in other neurodegenerative pathologies, such as Alzheimer’s disease, disturbances occur in vertical saccades by pathological changes in the riMLF and INC [42]. From our knowledge, this the first comprehensive study evaluating the horizontal and vertical DEM performance in MS patients in comparison to the performance of an age-matched control group with normal vision.

In the present work, since no quantifiable assessment of saccades was performed by objective methods, it is not possible to relate worse DEM scores exclusively to a specific saccadic impairment. Moreover, the authors as Tanke et al. [28] studied the relation between the DEM test and saccadic variables in children measured by an eye-tracker, and found no correlation between the DEM scores with objective saccadic performance, suggesting that the DEM test depends much more on fixation durations than saccades. Additionally, these authors found a strong positive correlation with visual processing speed [28]. In the case of subjects with MS, a reduced visual processing speed has been found in a high percentage of patients [43], but also fixation stability alterations [14] that affect fixation durations, and could be the cause of the reduced DEM performance.

The DEM test also integrates cognitive, processing, and verbal functions [22,44], abilities which have been reported to be impaired in MS patients [45]. Recently, two modifications of the DEM have been proposed to assess adults: the Adult Developmental Eye Movement (ADEM) Test [46] or its modification (which requires greater attention), the ADEMd [47]. These tests include two-digit numbers in order to increase the difficulty of the task, and, consequently, an increase in the cognitive visual-verbal demand. The results of the present paper suggest that the higher the cognitive demand, the higher will be the affection in MS subjects. In order to evaluate the cognitive factors in greater depth, it would be interesting to analyze whether an increase in difficulty could affect the results of the DEM test in future studies.

Regarding the MSON group, previously resolved optic neuritis does not seem to affect the ability to name numbers, neither vertical nor horizontally. The reason would be the same as discussed above for binocular summation. On the other hand, a slightly higher percentage of subjects in the MSON group showed a Type III classification (suggesting difficulties in visual-verbal skills) compared to the other groups, whereas for the Type I classification (representing normal results) this percentage was slightly lower in comparison to the others. Although these differences were not statistically significant, the results suggest a tendency that may be interesting to study in larger samples.

One remarkable finding was that a small percentage of patients in all groups that could not be classified according to the ratio. These patients showed vertical times that were higher than horizontal times, resulting in a lower-than-normal ratio. These results suggest that the ability to perform vertical cards may be more affected, and will be interesting to analyze this vertical component more specifically in future studies.

## 5. Conclusions

Subjects with MS spent more time than healthy subjects in performing K-D and DEM pseudo-reading tasks. The presence of a previous episode of ON does not seem to significantly affect the results when VA was restored and binocular summation is present. These tests can capture impairment of attention, language, and other areas that correlate with suboptimal brain function in addition to the oculomotor dysfunctions present in subjects with MS.

## Figures and Tables

**Table 1:** Clinical and demographic characteristics of the three study groups. Mean ± standard deviation (minimum–maximum) were reported. M = men; W = women. VA difference between eyes (ΔVA) and binocular VA for near vision were represented in log MAR. Negative sign (-) of heterophoria represent exophoria. The logarithm value of the stereopsis threshold was presented. Statistically significant differences were marked with an asterisk (*).

Groups (*n*)	Control (20)	MS (35)	MSON (30)	*p*
Sex	7 M, 13 W	9 M, 26 W	8 M, 22 W	0.43
Age (years)	51 ± 9	51 ± 10	50 ± 9	0.79
ΔVA (log MAR)	0.01 ± 0.05	0.01 ± 0.05	0.01 ± 0.04	0.62
Binocular Near VA (log MAR)	−0.03 ± 0.04	0.00 ± 0.09	0.01 ± 0.08	0.06
Heterophoria Near (Δ)	−3 ± 8	−3 ± 6	−2 ± 11	0.42
Stereopsis (log threshold)	1.66 ± 0.08	2.18 ± 0.52	2.80 ± 1.29	<0.05 *

**Table 2:** Median (and interquartile range) of the three study groups for K-D test. Minimum and maximum values were represented in square brackets. Statistically significant differences were marked with an asterisk (*).

K-D Test				
Groups (*n*)	Control (20)	MS (35)	MSON (30)	*p*
Time Card 1 (s)	18 (5) (10–22)	19 (6) (12–36)	21 (11) (13–45)	0.22
Errors	0 (0) (0–0)	0 (0) (0–2)	0 (0) (0–1)	0.54
Time Card 2 (s)	19 (5) (10–22)	19 (7) (11–42)	21 (10) (12–44)	0.11
Errors	0 (0) (0–0)	0 (0) (0–2)	0 (0) (0–5)	0.11
Time Card 3 (s)	16 (3) (14–21)	19 (8) (12–44)	21 (10) (13–43)	<0.05 *
Errors	0 (0) (0–0)	0 (0) (0–5)	0 (0) (0–2)	0.10
Time Total (s)	55 (11) (36–65)	56 (22) (36–120)	62 (33) (38–123)	<0.05 *

**Table 3:** Median (and interquartile range) of the three study groups for DEM test. Minimum and maximum were represented in square brackets. Statistically significant differences were marked with an asterisk (*).

DEM				
Groups (*n*)	Control (20)	MS (35)	MSON (30)	*p*
Time V1 (s)	16 (2) (12–19)	17 (5) (11–32)	20 (10) (12–32)	0.10
Errors V1	0 (0) (0–1)	0 (0) (0–1)	0 (0) (0–1)	0.91
Time V2 (s)	16 (2) (11–19)	17 (7) (11–34)	20 (8) (12–31)	0.06
Errors V2	0 (0) (0–0)	0 (0) (0–2)	0 (0) (0–2)	0.10
Total V Time (s)	32 (4) (24–37)	34 (12) (23–66)	40 (17) (24–62)	0.06
Time H (adj) (s)	32 (6) (22–40)	37 (14) (23–107)	41 (17) (21–92)	<0.05 *
Errors H	0 (0) (0–0)	0 (1) (0–6)	0 (0) (0–3)	0.09
Time Total (s)	64 (7) (49–76)	72 (26) (46–172)	80 (33) (49–149)	<0.05 *
Ratio	1.0 (0.11) (0.8–1.19)	1.09 (0.15) (0.93–1.62)	1.03 (0.25) (0.76–1.63)	<0.05 *
